# First report on the metabolic characterization of Sterigmatocystin production by select *Aspergillus* species from the *Nidulantes* section in *Foeniculum vulgare*

**DOI:** 10.3389/fmicb.2022.958424

**Published:** 2022-08-26

**Authors:** Pranab Kumar Mahata, Regina Sharmila Dass, Lokanadhan Gunti, Pooja Appasaheb Thorat

**Affiliations:** Fungal Genetics and Mycotoxicology Laboratory, Department of Microbiology, School of Life Sciences, Pondicherry University, Puducherry, India

**Keywords:** *Aspergillus*, sterigmatocystin (STC), food spoilage, mycotoxigenic, regulatory genes, HPLC

## Abstract

Spices are typically grown in climates that support the growth of toxigenic fungi and the production of mycotoxins. The *Aspergilli* described in this study, as well as the sterigmatocystin (STC) detected, are causes for concern due to their potential to induce food poisoning. One of the most well-known producers of the carcinogenic STC is *Aspergillus nidulans*. This research explores the occurrence of STC-producing fungi in *Foeniculum vulgare*, a spice that is marketed in India and other parts of the world. This innovative study details the mycotoxigenic potential of five *Aspergilli* belonging to Section *Nidulantes*, namely *Aspergillus latus* (02 isolates), *Emericella quadrilineata* (02 isolates), and *Aspergillus nidulans* (01 isolate), with respect to STC contamination. These five isolates of *Aspergilli* were screened to produce STC on yeast extract sucrose (YES) medium in a controlled environment with regard to light, temperature, pH, and humidity, among other variables. The expression patterns of regulatory genes, namely, *aflR*, *laeA*, *pacC*, *fluG*, *flbA*, *pksA*, and *mtfA* were studied on the Czapek–Dox agar (CDA) medium. STC biosynthesis by the test isolates was done in potato dextrose broth (PDB) under optimum conditions, followed by the extraction and purification of the broth using ethyl acetate. High-performance liquid chromatography (HPLC) with an ultraviolet (UV) detector was utilized to detect compounds in eluted samples. *F. vulgare* contains *Aspergilli* that have been shown to have mycotoxigenic potential, which can accumulate in the spice during its active growth and thereby cause the elaboration of mycotoxins.

## Introduction

*Aspergillus* is a genus of filamentous fungi that has grown to comprise more than 250 different mold species (Houbraken and Samson, 2011) across the world since it was first described approximately 300 years ago. Some species of these mitosporic, conidial fungi reveal teleomorphic forms and are hence classified as part of the phylum Ascomycota (Bennett, 2010). *Aspergillus* species are extensively employed by the medical (Bladt et al., 2013) and industrial sectors (Polizeli et al., 2016) for the applications of their beneficial secondary metabolites. However, certain *Aspergillus* species are pathogenic to humans (Alshehri and Palanisamy, 2020), animals, and birds (Arné et al., 2021) and are also prolific producers of secondary metabolites known as mycotoxins (Navale et al., 2021).

As the name suggests, mycotoxins are highly toxic and produced predominantly by fungi such as *Aspergillus*, *Penicillium*, and *Fusarium* (Liew and Mohd-Redzwan, 2018). They are frequently discovered in various foods or feedstuffs, thereby posing a significant risk to human and animal health, as well as economic losses caused by STC contamination (Cardwell et al., 2001). This is a major food safety issue for underdeveloped countries, where detection, monitoring, and regulating procedures are often lacking to protect the food supply from mycotoxins. The genes required for secondary metabolite production in fungi are arranged in gene clusters that encode key enzymes (Keller et al., 2005), such as polyketide synthases, non-ribosomal polypeptide synthetases, terpene cyclases, dehydrogenases, esterases, and methyltransferases (Brakhage, 2013). Though gene clusters are of enormous biological and economic importance, information about their structure, function, and regulation remains unclear.

The flowering plant *Foeniculum vulgare* Mill, often known as fennel or saunf in Hindi, is a member of the Umbelliferae (Apiaceae) family. It grows in the United States, Northern Europe, Southern Canada, Asia, and Australia. It is a good source of protein, fiber, vitamin A, thiamin, vitamin C, and minerals, namely, calcium, iron, magnesium, manganese, and fatty acids. Essential oils from fennel are utilized in beverages, bread, pickles, pastries, and cheese. Well known is their antioxidant, cryoprotective, estrogenic, hyperglycemic, and hyper-protective properties (Javidnia et al., 2003; Samadi-Noshahr et al., 2021). In addition, they contain anti-inflammatory and anti-tumor properties (Malini et al., 1985) and are used to treat bacterial, viral, fungal, mycotic, and protozoan infections (Rather et al., 2016). Furthermore, there is evidence that fennel may offer protection against cardiovascular disease (Osman et al., 2017).

The annual fennel production in India was 58,265 tons in 2010–2011 (Abubacker, 2011) and has increased to 139,760 tons during 2019–2020 (Anonymous, 2020a), indicating that consumption is soaring. Fennel is grown in India and exported to other nations. In 2018 and 2020, the range of exports was between 2,35,62,460 and 2,02,95,380 million metric tons (Anonymous, 2020b). The Ministry of Food Processing estimates that poor scientific conditions, unhygienic harvesting practices, and post-harvest procedures cost 93,000 crores of Indian rupees (INR) in economic losses, of which fennel is one (Moloney, 2019).

*F. vulgare*, like the majority of cereals and grains, is susceptible to infection by a variety of mycoflora, some of which can severely reduce the crop’s economic worth. However, fungi from the *Aspergillus* and *Penicillium* genera can thrive in low humidity and cause significant damage to crops. Infections caused by *Aspergillus* spp. and several other species have been of particular concern in recent years, as all these fungal phytopathogens release hazardous mycotoxins that cause substantial contamination of food (Navale et al., 2021), feed (Variane et al., 2018), and agricultural goods, namely, spices (Garcia et al., 2018). Spices (e.g., fennel seeds) serve as an important ingredient in Indian cuisine (Siruguri and Bhat, 2015). Toxic secondary metabolites produced by contaminating mycoflora have a negative influence on their quality.

Aflatoxins (AFs) are the most extensively researched mycotoxins, and are primarily synthesized by *Aspergillus flavus* and *Aspergillus parasiticus.* It has been shown to be harmful to fetuses (Lauer et al., 2019), possibly teratogenic, and the most carcinogenic substance in the world, according to the International Agency for Research on Cancer (IARC). STC is a highly mutagenic chemical that, in substantial doses, is also a recognized liver carcinogen. The IARC classified STC as a group 2B carcinogen, indicating that it may cause cancer in people. No country has defined a maximum permissible level for it because of its infrequent natural occurrence, even though it is exceedingly hazardous.

Studies on the model organism *Aspergillus nidulans*, which produces the precursor of aflatoxin, namely STC, have led to a greater understanding of the metabolic pathways involved in the formation of AF (Brown et al., 1996). *A. nidulans* reproduces predominantly through the formation of asexual spores called conidia and produces the mycotoxin STC, the penultimate precursor in the AF biosynthetic process, which is found in related organisms like *A. parasiticus*, *A. flavus*, and *Aspergillus nomius* (Cole and Cox, 1981). Both polyketides are known for causing mammalian hepatocarcinoma (Bressac et al., 1991), animal toxicity (Anderson et al., 1990; Balogh et al., 2019) and are believed to be immune impairing to infants as well as the aged (Cardwell and Miller, 1996). It has been proposed that the STC potentially plays a part in the development of chronic liver disease in human beings in Africa (Cole et al., 2003). The toxic effect of STC is modest, but the primary issue is that it can cause cancer; the carcinogenicity is approximately one-tenth that of AFB1, which is a significant difference. STC structure is similar to AFs, in that it is made up of a xanthone nucleus that is connected to a bisfuran ring structure. STC is soluble in acrylonitrile, benzene, chloroform, ethyl acetate, and acetone. However, it is insoluble in water and petroleum ether. Many *Aspergillus* species, namely, *A. versicolor*, *A. nidulans*, *A. sydowii*, and various species of *Bipolaris*, generate STC. Mycotoxin gene clusters from *A. nidulans* and *A. parasiticus* exhibit a high degree of homology (Yu et al., 2004), which aids in the study of AF/STC synthesis and regulation measures. In *A. nidulans*, the expression of STC is controlled by an intricate network of genes that are clustered together on chromosome IV around a 60 kb DNA region. The cluster contains 25 genes that are involved in the intricate processes necessary for the STC pathways to function properly (Brown et al., 1996).

*Aspergillus* diversity, phylogeny, and mycotoxin-producing potential in *F. vulgare* have not been previously reported by researchers. In a recent research article (Mahata et al., 2022), a detailed morphological identification of *Aspergilli*, supported by molecular barcoding using ITS 1 and ITS 4 as well as β-tubulin as genetic markers (Tam et al., 2014), has been published. Accurate identification of *Aspergilli* has bridged the information gap on misidentification and determining the STC-producing potential, to recognize the significant danger associated with the consumption of toxin-laden food and feed in humans and animals, respectively.

This study aims to provide knowledge on the prevalence of *Aspergillus* species producing STC, associated with fennel. *Aspergilli* were examined on a mycological medium to identify STC-producing isolates in the preliminary screening procedure. The identification of genes involved in the synthesis of toxins and the purification of mycotoxin in the laboratory allowed us to assess the potential toxigenicity of these species. As a result, this article includes an inventory of potentially STC-producing *Aspergillus* species belonging to the *Nidulantes* Section of the *Aspergillus* taxonomy.

## Materials and methods

### Isolates of fungal species

In our analyses (Mahata et al., 2022), we procured 70 samples of an Indian spice, *F. vulgare* from several retail marketplaces around India, namely, New Delhi, Lucknow, Pinjore, West Midnapore, and Puducherry. The mycofloral tests of Indian fennel samples were carried out using standard agar plating techniques defined by the International Seed Testing Association (ISTA). *Aspergillus* isolates were screened by plating *F. vulgare* spice samples on mycological media that included potato dextrose agar (PDA) and Czapek–Dox agar (CDA) (HiMedia^®^, Mumbai, India) by adopting techniques described by researchers (Xie et al., 2007; Hamzah et al., 2018). All the fungal flora of fennel samples were isolated and identified using standard fungal manuals and keys (Thom and Raper, 1945; Refai et al., 2014). A single-point or three-point inoculation technique was used to inoculate the isolates into glass Petri plates (100 × 15 mm, S-line, Borosil^®^, Mumbai, India). After 7 days of incubation, colony sizes, coloration, textures, spore germination pattern, obverse and reverse colonial color schemes, and the presence or absence of ascomata (in older cultures kept for 15–25 days) were observed. Besides this, the study of hyphal features such as septation, vesicle morphology, conidiophore architecture, sterigmata arrangement, and conidia development received special attention. These microscopic characteristics of individual *Aspergilli* were studied consecutively using light microscopy (Olympus CH20i), scanning electron microscopy (SE; Hitachi, Model E-1010), and differential interference contrast (DIC; Nikon Upright Motorized Microscope, ECLIPSE Ni series, Nikon Corporation, Tokyo) microscopy. By examining microscopic characteristics as described in identification keys and manuals, standard protocols were adopted (Thom and Raper, 1945; Varga and Samson, 2008). Light and Scanning electron micrographs (SEM) were acquired at the Fungal Genetics and Mycotoxicology Laboratory, Department of Microbiology and Central Instrumentation Facility (CIF), Pondicherry University, while DIC micrographs were documented at the Fungal Biotechnology Laboratory, Department of Biotechnology, School of Life Sciences, Pondicherry University.

**FIGURE 1:**
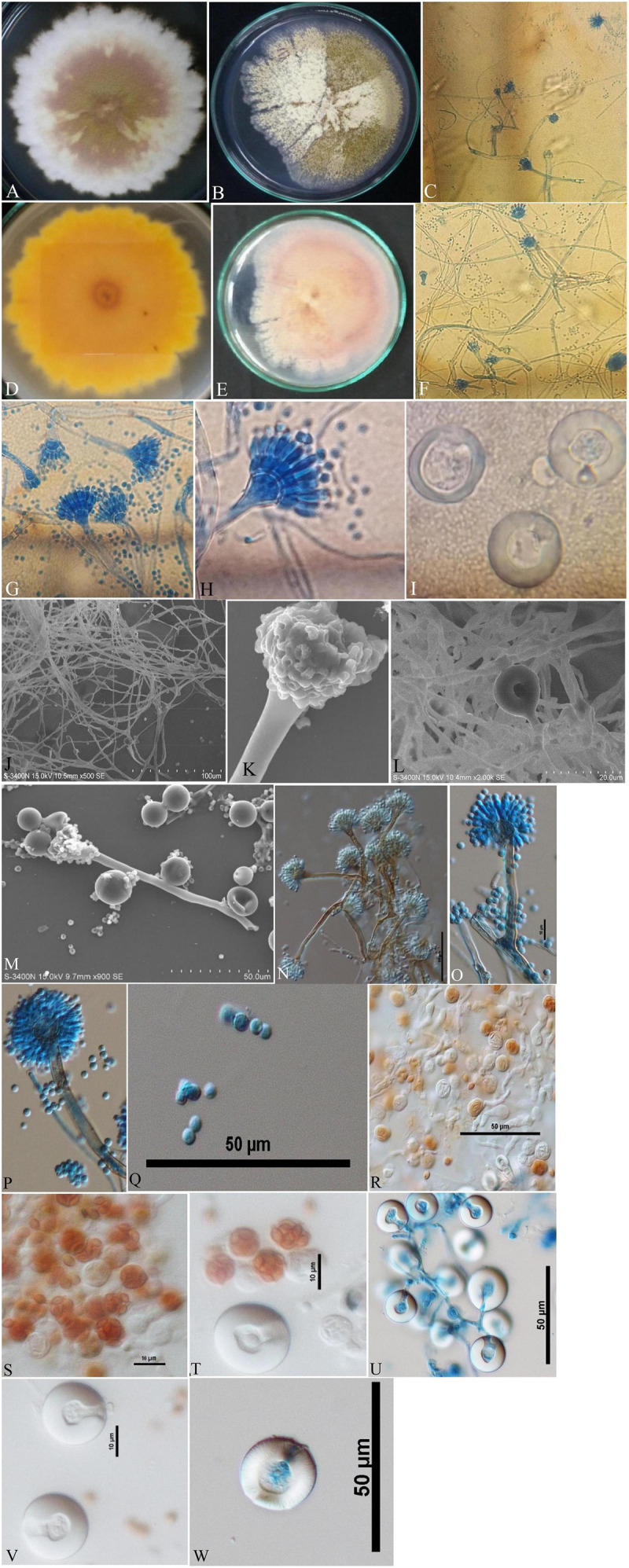
*Aspergillus nidulans* (Acc. No. MN791101) colonies, conidiophores, vesicles, metulae, phialides, conidia, asci and Hülle cells. **(A,B)** (Obverse) Colonies cultured at 25°C on PDA and CDA, respectively; **(C)** (CLM) hyphae and conidiophores; **(D,E)** (Reverse) colonies on PDA and CDA, respectively; **(F,G)** (CLM) magnified view of conidiophores in development; **(H)** (CLM) conidiophore enlarged; **(I)** (CLM) Hülle cells; **(J)** (SEM) mycelium, conidiophores and developing spores; **(K)** (SEM) young developing conidial head; **(L)** (SEM) mycelium and Hülle cell development; **(M)** (SEM) Hülle cells; **(N–P)** (DIC) smooth walled, sinuate conidiophores and columnar conidial heads with small, hemispherical vesicles, metulae, phialides and conidia; **(Q)** (DIC) rough, globose conidia; **(R–T)** (DIC) asci; and **(U–W)** (DIC) Hülle cells.—Scale bars: **(J)** = 100 μm; **(M,N,P–R,U,W)** = 50 μm; **(K,L)** = 20 μm; **(O,S,T,V)** = 10 μm. **(A–D,H,I,K–M,P,Q,T,W)** were published in the first and correspondence authors’ recent research article (Mahata et al., 2022).

**FIGURE 2:**
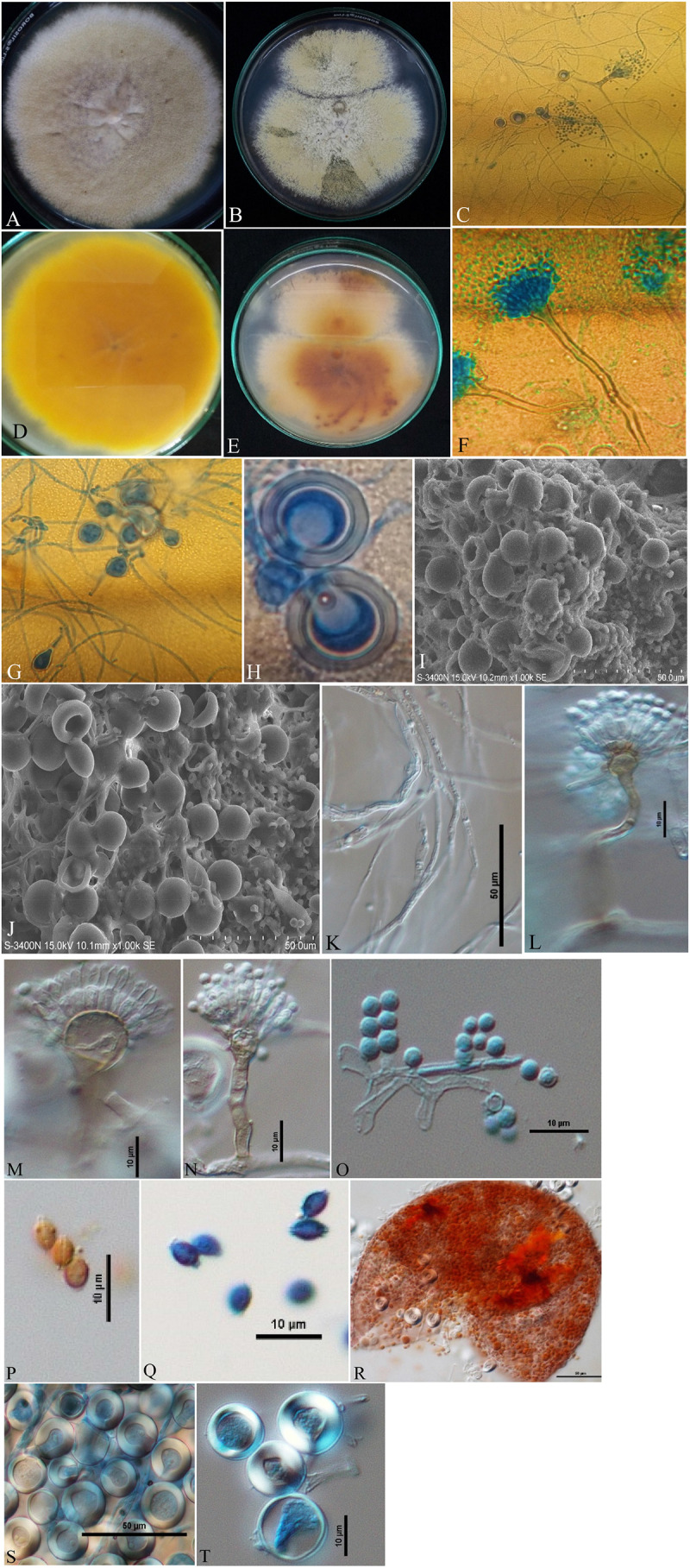
*Aspergillus quadrilineatus/Emericella quadrilineata* (Acc. No. MN791105) colonies, conidiophores, vesicles, metulae, phialides, conidia, cleistothecia, ascospores and Hülle cells. **(A,B)** (Obverse) Colonies were cultured at a temperature of 25°C on PDA and CDA; **(C)** (CLM) conidiophores and development of spores; **(D,E)** (Reverse) colonies on PDA and CDA, respectively; **(F)** (CLM) conidiophores; **(G,H)** (CLM) the formation of Hülle cells and Hülle cells; **(I,J)** (SEM) formation of Hülle cells; **(K)** (DIC) mycelium growth; **(L**,**M)** (DIC) conidiophores with smooth, sinuate margins and short, columnar conidial heads with hemispherical vesicles, metulae, phialides, and conidia; **(O)** (DIC) conidia; **(Q)**, (DIC) ascospores having lenticular, smooth-walled ascospores; **(P)** (DIC) smooth-walled lenticular (unstained with cotton-blue) ascospores; **(R)** (DIC) cleistothecium ruptured; and **(S,T)** (DIC) Hülle cells.—Scale bars: **(I–K,R,S)** = 50 μm; **(L–Q,T)** = 10 μm. **(A,D,F,H,J,M,O,P,R–T)** Were published in the first and correspondence authors’ recent research article (Mahata et al., 2022).

**FIGURE 3:**
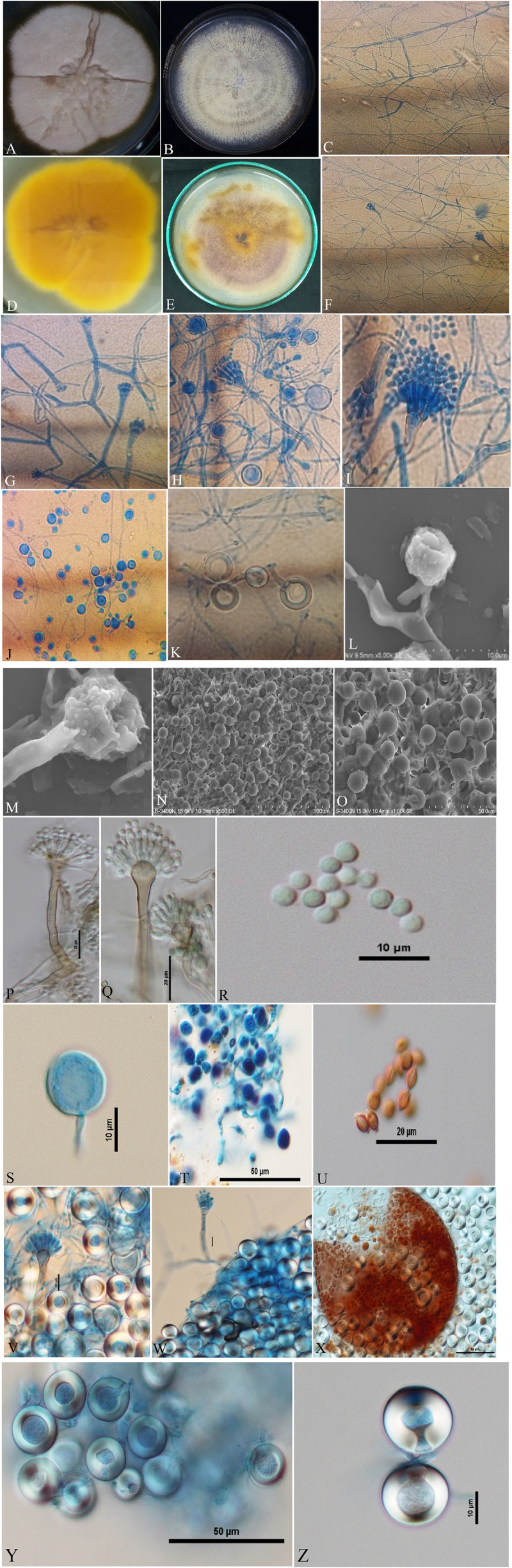
*Aspergillus latus* (Acc. No. MN791110) colonies, conidiophores, vesicles, metulae, phialides, conidia, cleistothecia, asci, ascospores and Hülle cells. **(A,B)** (Obverse) Colonies cultured at 25°C on PDA and CDA, respectively; **(C)** (CLM) hyphae and magnified view of conidiophores in development; **(D,E)** (Reverse) colonies on PDA and CDA, respectively; **(F**–**H)** (CLM) the growth of conidiophores and spores; **(I)** (CLM) conidiophore; **(J,K)** (CLM) Hülle cells; **(L,M)** (SEM) conidiophores; **(N,O)** (SEM) Hülle cells; **(P,Q)** (DIC) smooth walled, sinuate conidiophores and columnar conidial heads with small, hemispherical vesicles, metulae, phialides and conidia; **(R,S)** (DIC) conidia with a globose appearance; **(T)** (DIC) asci bearing ascospores; **(U)** (DIC) ascospores lenticulares; **(V,W)** (DIC) Hülle cells; **(X)** (DIC) cleistothecium ruptured, having asci, and enclosed by Hülle cells; and **(Y,Z)** (DIC) Hülle cells.—Scale bars: **(L–N)** = 100 μm; **(O,T,X,Y)** = 50 μm; **(P,Q,U)** = 20 μm; **(L,R,S,V,W,Z)** = 10 μm. **(A,D,I,K,M,Q,R,T,U,V,X,Z)** Were published in the first and correspondence authors’ recent research article (Mahata et al., 2022).

Isolates of *A. nidulans* (Figure 1), *E. quadrilineata* (Figure 2), and *A. latus* (Figure 3) were subjected to in-depth research pertaining to the properties of their macromorphological and micromorphological structures as required in fungal taxonomic studies. Fungal stock cultures were prepared and maintained at –20°C. These macromorphological features include 7-day colony development, coloration, and textures on PDA and CDA media. Micromorphological considerations, moreover, included microscopic analysis of asexual and sexual phases of the *Aspergillus* life cycle.

The five *Aspergillus* isolates (Table 1) included in this study (*A. nidulans-*01 isolate; *E. quadrilineata-*02 isolates; *A. latus-*02 isolates) were isolated and identified by morphological and molecular methods using the internal transcribed spacer (ITS) and β-tubulin marker genes. The isolates inspected were from one of our previously published research studies (Mahata et al., 2022). Additionally, cultural features of *E. quadrilineata* and *A. latus* on the Czapek–Dox agar medium, as well as microscopic images of *A. nidulans*, *E. quadrilineata*, and *A. latus*, have been shown to aid in the morphological identification process.

**TABLE 1:** *Aspergilli* used in this study, ITS and β-tubulin gene sequences GenBank accession numbers.

Sl. No.	FGM lab isolate code	Genus/species	Accession number (ITS)	Accession number (β-tubulin)
1	45	*A. nidulans*	MN309877	MN791101
2	49	*E. quadrilineata*	−	MN791104
3	58	*E. quadrilineata*	−	MN791105
4	4	*A. latus*	−	MN791110
5	11	*A. latus*	−	MN791111

The ITS gene represents the Internal Transcribed Spacer.

FGM Laboratory-Fungal Genetics and Mycotoxicology Laboratory; Pranab Kumar Mahata and Regina Sharmila Dass identified, cultivated, and analyzed all fungal isolates.

### Screening for secondary metabolites in *Aspergillus nidulans, Emericella quadrilineata*, and *Aspergillus latus*

#### Media

In the preliminary, each *Aspergillus* isolate was inoculated in yeast extract sucrose (YES) (HiMedia^®^, Mumbai, India) agar culture medium, and a secondary metabolite production assay for STC was performed.

#### Cultivation and fluorescence detection

All of the *Aspergillus* isolates that had been evaluated for STC production were cultured in YES medium, incubated for the appropriate incubation time, and assessed by UV fluorescence detection (Fente et al., 2001) to confirm the presence of STCs. The experiment was carried out by adding 2% β-cyclodextrin (HiMedia^®^, Mumbai, India) to the medium. The plates were incubated at 28°C for 4 days. Upon examination using ultraviolet radiation (365 nm), the development of fluorescence in the agar medium around the *Aspergillus* colonies was indicative of a preliminary positive result. All experiments were carried out in triplicates. For the demonstration of their STC-producing abilities, all isolates were grown in the CDA medium to determine their gene expression profiles.

### RNA-Seq and gene expression analysis

#### Conditions of growth

Each *Aspergillus* isolate was cultivated using the CDA medium designed for STC-induction for a set period of time, typically 7 days.

#### RNA total extraction

High-quality RNA was isolated using the Qiagen RNeasy kit (QIAGEN India Pvt. Ltd., New Delhi, India) method (following the manufacturer’s instructions). In order to avoid genomic DNA contamination, DNase treatment and purification processes were performed. RNA was measured using UV-Vis, and its quality was tested using 260/280, which showed that the purity was satisfactorily good (1.8–2). The RNAs were also run on a 1% agarose gel, to assess their purity.

### Polymerase chain reaction primers

#### Reverse transcription-polymerase chain reaction and polymerase chain reaction-amplified gene expression

cDNA was produced utilizing the Bio-Rad iScript cDNA synthesis kit (Bio-Rad Laboratories India Pvt. Ltd., Haryana, India) (per the manufacturer’s instructions) using random hexamers and oligo(dT) primers as follows: 5X Mix 10, 18 μL of nuclease-free water, 2 μg RNA in 10 μL, and 2 μL of reverse transcriptase. In a PCR cycler, the sample was incubated as follows: 5 min of priming at 25°C, 20 min of RT at 46°C, and 1 min of RT inactivation at 95°C.

**FIGURE 4:**
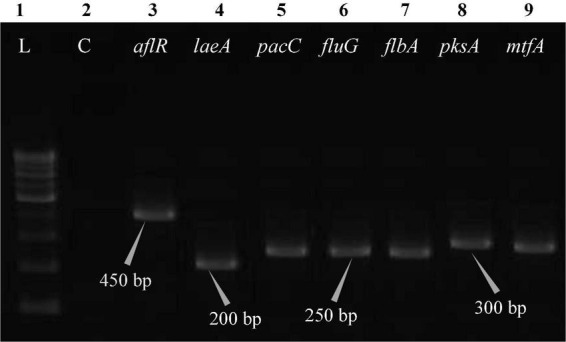
Electrophoretogram showing PCR amplification of transcribed genes in *A. nidulans* (MN791101). Lane 1–100 bp DNA ladder, Lane 2-Negative control PCR, Lane 3 through 9 includes amplicons for the *aflR*, *laeA*, *pacC*, *fluG*, *flbA*, *pksA*, and *mtfA* genes, sequentially.

**FIGURE 5:**
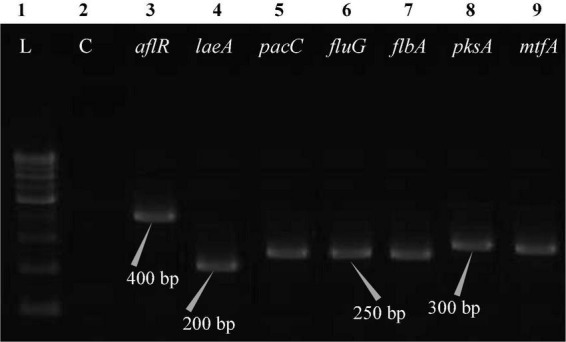
Electrophoretogram showing PCR amplification of transcribed genes in *E. quadrilineata* (MN791104). Lane 1–100 bp DNA ladder, Lane 2-Negative control PCR, Lane 3 through 9 includes amplicons for the *aflR*, *laeA*, *pacC*, *fluG*, *flbA*, *pksA*, and *mtfA* genes, sequentially.

**FIGURE 6:**
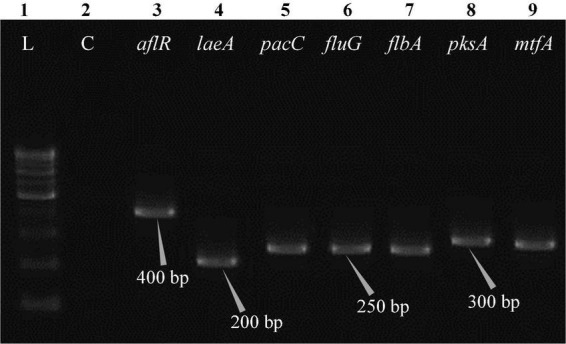
Electrophoretogram showing PCR amplification of transcribed genes in *E. quadrilineata* (MN791105). Lane 1–100 bp DNA ladder, Lane 2-Negative control PCR, Lane 3 through 9 includes amplicons for the *aflR*, *laeA*, *pacC*, *fluG*, *flbA*, *pksA*, and *mtfA* genes, sequentially.

**FIGURE 7:**
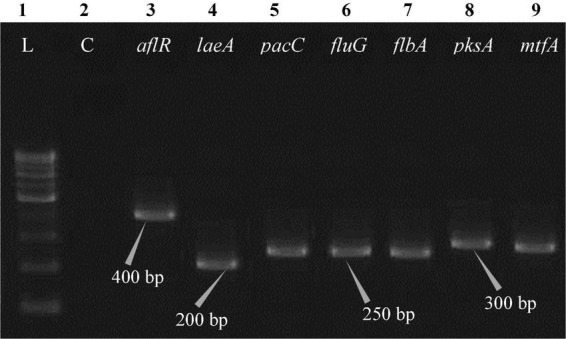
Electrophoretogram showing PCR amplification of transcribed genes in *A. latus* (MN791110). Lane 1–100 bp DNA ladder, Lane 2-Negative control PCR, Lane 3 through 9 includes amplicons for the *aflR*, *laeA*, *pacC*, *fluG*, *flbA*, *pksA*, and *mtfA* genes, sequentially.

**FIGURE 8:**
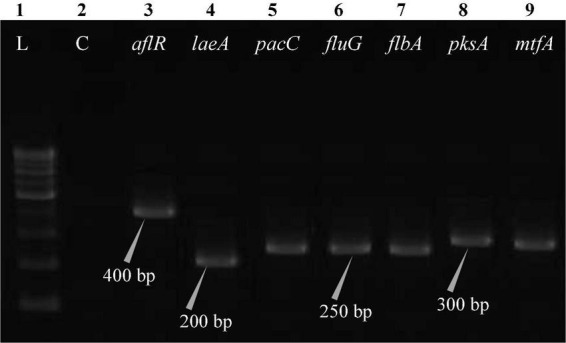
Electrophoretogram showing PCR amplification of transcribed genes in *A. latus* (MN791111). Lane 1–100 bp DNA ladder, Lane 2-Negative control PCR, Lane 3 through 9 includes amplicons for the *aflR*, *laeA*, *pacC*, *fluG*, *flbA*, *pksA*, and *mtfA* genes, sequentially.

The polymerase chain reaction (PCR) was performed on a Bio-Rad thermocycler (Bio-Rad Laboratories India Pvt. Ltd., Haryana, India) using all seven primers (Table 2) for the *Aspergillus* isolates. The reaction volumes for real-time polymerase chain reaction (real-time PCR) were fixed at 25 μL, which contained 2X PCR Master Mix (12.5 μL; Thermo Scientific™, Vilnius, Lithuania), forward primer (1 μL), reverse primer (1 μL), cDNA (1 μL; 50 ng RNA equivalent cDNA was used), and 9.5 μL of molecular biology grade water (9.5 μL; HiMedia^®^, Mumbai, India). The cycle protocol included a 5 min denaturation step at 95°C, 40 cycles of denaturation at 95°C for 10 s, annealing at 55°C for 20 s, and extension at 72°C for 1 min, following the final extensions step for 10 min at 72°C. To validate adequate PCR amplification (Figures 4–8), the sizes of select isolates related to STC-expression genes were determined using a 1% agarose gel and a 100 bp DNA ladder (Thermo Fisher Scientific, Vilnius, Lithuania).

**TABLE 2:** Primers sequences used.

Sl. No.	Gene	Primer sequence (5′–3′)	Amplicon size (bp)	References
1	*aflR*	F: GCCATCCTGTCTCCGAATAC R: CGAACCTCTACGACTGTCTTG	450	Delgado-Virgen and Guzman-de-Peña, 2009
2	*laeA*	F: GGTGACGATTTGTATAGTCC R: CTCTTCATGAAACTGGTTTC	200	Delgado-Virgen and Guzman-de-Peña, 2009
3	*pacC*	F: GACTGACGGTATGACTTCTG R: GTTGGCAATGTAGTTACGTA	250	Delgado-Virgen and Guzman-de-Peña, 2009
4	*fluG*	F: ACCCTAATGTTTATTTGGAT R: TGGATAGGTCTGGTATAAGG	250	Delgado-Virgen and Guzman-de-Peña, 2009
5	*flbA*	F: TCCCTCAAATTCTCTCAATCGAACCGG R: GTAGAATGACAGGTTTTCTTCGCAGA	250	Delgado-Virgen and Guzman-de-Peña, 2009
6	*pksA*	F: TTCTGCATGGGTTCCTTGGC R: CCATTGTGGGCCGGTAAACA	300	Leema et al., 2011
7	*mtfA*	F: GCCCTCACCCTCATCGGCAATG R: GGTCGTGGTTCTGCTGGTAGGGTGT	250	Ramamoorthy et al., 2013

#### Sterigmatocystin purification

All *Aspergillus* isolates were grown in a carbohydrate-rich medium (containing D-glucose at a concentration of 20 g/L and potato infusion at a concentration of 200 g/L) at 180 rpm on a rotary shaker (VDRL, Delhi, India) for 13 days, with a temperature of 23°C. The cultures (2L) were filtered in order to separate the mycelium from the broth. The extraction and purification methods were adopted as given by Han et al. (2020). The ethyl acetate was added to the filtered broth and mycelium, followed by evaporation in a fume hood (Ganapathy Industries, Karnataka, India) to maintain the requisite level of dryness. The crude ethyl acetate extract of broth (12.8 g) was then separated through a silica gel column and eluted using a gradient of petroleum ether/acetone (in 50:1–1:1, v:v) and dichloromethane (from 50:1 to 1:1, v:v). The eluted samples were pooled for HPLC analysis.

#### High-performance liquid chromatography with ultraviolet detection

The use of reverse phase high-performance liquid chromatography (RP–HPLC) was employed in order to investigate the production of STC. The HPLC system with an ultra-violet (UV) detector at 325 nm wavelength (Shimadzu Corporation in Kyoto, Japan) and the stationary phase, C18 reverse phase column (Thermo Fisher Scientific, Vilnius, Lithuania), 150 mm × 4.6 mm, 3 μm particle size, was utilized. Each sample, including the STC standard, was produced in an acetonitrile solution. The separation was achieved by chromatographic conditions, i.e., an isocratic mobile phase of acetonitrile: Water (60:40) with a flow rate of 1.0 mL min^–1^, column oven temperature of 30°C, an injection volume of 10 μl, and a total run time of 30.0 min. The retention time (t_*R*_) of the STC standard was determined to be 9.982 min. The STC in samples was confirmed by comparing t_*R*_ values with those of the standard.

## Results

The analysis of 70 different samples of *F. vulgare* led to the isolation and identification of 14 distinct species of *Aspergilli*, from across a number of geographical regions in India. A very high incidence of *Aspergillus niger* was recorded, followed by other *Aspergilli* species, namely *A. flavus, A. terreus*, *A. nidulans*, *A. tamarii*, *A. species*, *Emericella quadrilineata*, *A. fumigatus*, *A. latus*, *A. aureoterreus, A. awamori*, *A. brasiliensis*, *A. ochraceus*, and *A. sydowii*, in decreasing order. The above findings of a detailed phenotypic and anatomical characterization of *Aspergilli* and authentication using a molecular fungal barcode have been previously published (Mahata et al., 2022).

### Morphology and molecular biomarkers of *Aspergillus* species

Following the phenotypic studies, five *Aspergillus* isolates belonging to three distinct species categories were subjected to detailed genetic analysis using the ITS gene of the ribosomal DNA (rDNA) and for β-tubulin gene sequences for molecular identification. The *Aspergilli* with their GenBank accession numbers are presented (Table 1).

### Toxicity assessment

**FIGURE 9:**
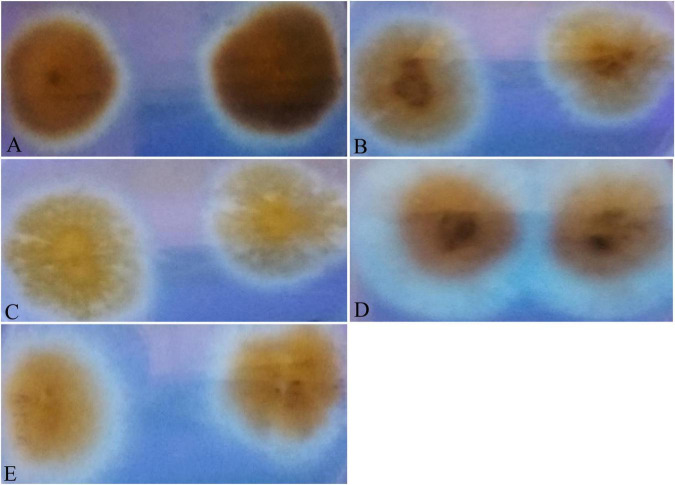
Fungal cultures of *Aspergilli* on yeast sucrose medium (YES), supplemented with β-methyl cyclodextrin, incubated at 25°C and photographed on the fourth day. The colonies of **(A)** *A. nidulans* (MN791101), **(B)** *A. latus* (MN791110), **(C)** *E. quadrilineata* (MN791105), **(D)** *E. quadrilineata* (MN791104), and **(E)** *A. latus* (MN791111) fluoresced with a light blue glow under long wavelength UV light.

All isolates were tested under UV light (365 nm) and the incubation duration was 4 days on YES agar supplemented with 2% β-cyclodextrin at 28°C. The result was evidently decisive, as fluorescence was detected after 4 days of incubation for all sterigmatocystin isolates that were tested (Table 3). The fluorescence displayed a light blue glow (Figure 9), which served as a preliminary tool for toxicity assessment. In parallel to this mycotoxin, other compounds produced by *Aspergillus* isolates can exhibit blue fluorescence when exposed to UV light; hence, in order to validate this hypothesis in our investigations, additional genomic profiling and STC purification tests were carried out.

**TABLE 3:** UV-visualization of *Aspergillus* isolates on β-cyclodextrin supplemented baseline culture media on the fourth day of incubation at 28°C.

Genus/species	Isolate/lab code	Fluorescence on 2% β-cyclodextrin supplemented media
*A. nidulans*	FOEVPRB45	Bright blue fluorescence
*E. quadrilineata*	FOEVPRB49	Bright blue fluorescence
*E. quadrilineata*	FOEVPRB58	Bright blue fluorescence
*A. latus*	FOEVPRB4	Bright blue fluorescence
*A. latus*	FOEVPRB11	Bright blue fluorescence

### Gene profiling

**FIGURE 10:**
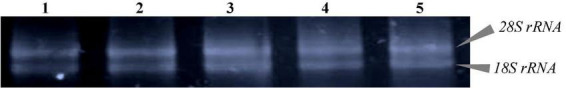
The agarose gel electrophoresis images show that the total RNA extracted from five different *Aspergilli* is comprised of two bright bands that correspond to *28S rRNA* and *18S rRNA*. Lane 1- *A. latus* (MN791111), Lane 2- *A. latus* (MN791110), Lane 3- *A. nidulans* (MN791101), Lane 4- *E. quadrilineata* (MN791104), and Lane 5- *E. quadrilineata* (MN791105).

Total RNA was obtained from all five test isolates of *Aspergillus* isolates. Agarose gel (1%) electrophoresis was performed in order to detect 28SrRNA and 18SrRNA as distinct bands (Figure 10). Seven genes of the STC biosynthesis cluster were detected using PCR to examine their role in toxin production. *A. latus* (02 isolates), *E. quadrilineata* (02 isolates), and *A. nidulans* (01 isolate) are all known to have the potential ability to produce STC among the species that were investigated. A positive amplification pattern (Table 4) for all seven genes tested was obtained for the *A. nidulans* FOEVPRB45 (Figure 4), *E. quadrilineata* FOEVPRB49 (Figure 5), *E. quadrilineata* FOEVPRB58 (Figure 6), *A. latus* FOEVPRB4 (Figure 7), and *A. latus* FOEVPRB11 (Figure 8) isolates in our inquiry. This led us to infer that a portion of the *Aspergillus* population has a significant potential to contaminate *F. vulgare* with STC in India.

**TABLE 4:** PCR amplification data for STC biosynthetic gene cluster.

Genus/species	*aflR*	*laeA*	*pacC*	*fluG*	*flbA*	*pksA*	*mtfA*
*A. nidulans* FOEVPRB45	+	+	+	+	+	+	+
*E. quadrilineata* FOEVPRB49	+	+	+	+	+	+	+
*E. quadrilineata* FOEVPRB58	+	+	+	+	+	+	+
*A. latus* FOEVPRB4	+	+	+	+	+	+	+
*A. latus* FOEVPRB11	+	+	+	+	+	+	+

+: Amplicon positive, with the expected size of the target gene.

### *Aspergillus* species sterigmatocystin assay

**FIGURE 11:**
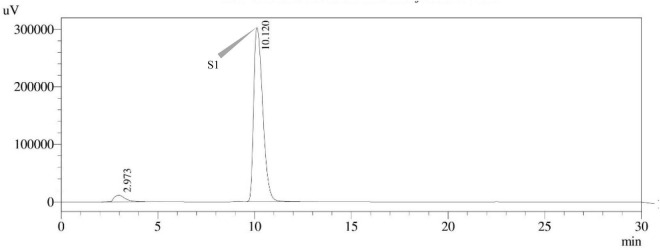
HPLC chromatogram of *A. nidulans* (MN791101) producing sterigmatocystin, detected using UV light at 325 nm with retention time (t_*R*_) of 10.120 min.

**FIGURE 12:**
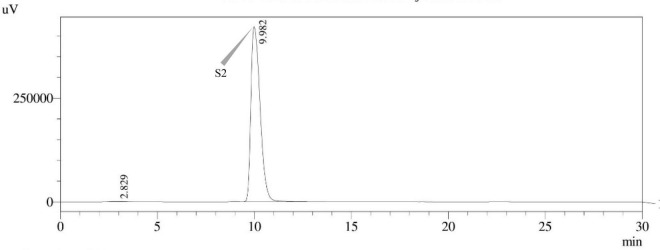
HPLC chromatogram of *E. quadrilineata* (MN791104) producing sterigmatocystin, detected using UV light at 325 nm with retention time (t_*R*_) of 9.982 min.

**FIGURE 13:**
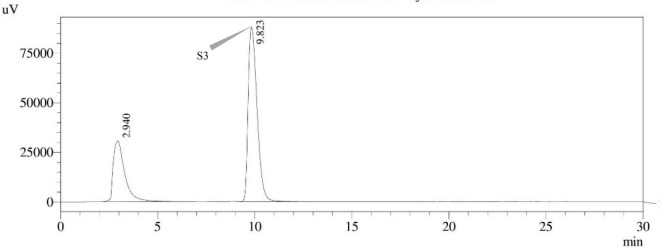
HPLC chromatogram of *E. quadrilineata* (MN791105) producing sterigmatocystin, detected using UV light at 325 nm with retention time (t_*R*_) of 9.823 min.

**FIGURE 14:**
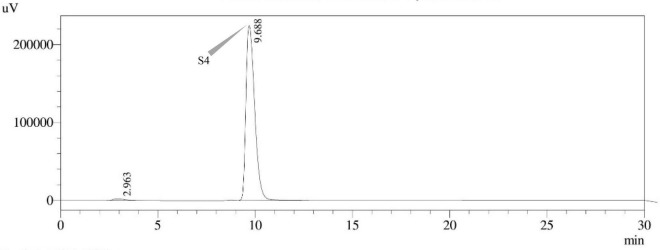
HPLC chromatogram of *A. latus* (MN791110) producing sterigmatocystin, detected using UV light at 325 nm with retention time (t_*R*_) of 9.688 min.

**FIGURE 15:**
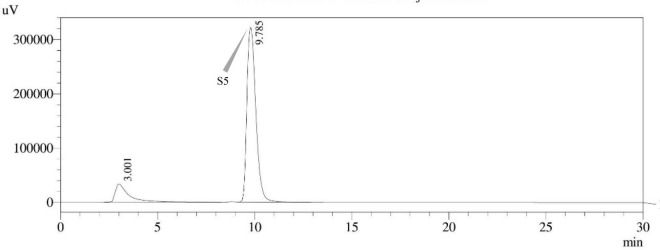
HPLC chromatogram of *A. latus* (MN791111) producing sterigmatocystin, detected using UV light at 325 nm with retention time (t_*R*_) of 9.785 min.

Sterigmatocystin was detected by an HPLC-UV assay at 325 nm. The retention time with the names of the peaks for the test isolates of *Aspergilli* is provided (Table 5). The retention time (t_*R*_) was 10.120 min for *A*. *nidulans* FOEVPRB45 and 9.688 min for *A. latus* FOEVPRB4 (Figures 11–15).

**TABLE 5:** Detection of sterigmatocystin using high-performance liquid chromatography (HPLC) with UV light at 325 nm and retention time (t_*R*_) in minutes for the investigated isolates.

Genus/species	Name of peak	Retention time (min)
*A. nidulans* FOEVPRB45	S1*	10.120
*E. quadrilineata* FOEVPRB49	S2*	09.982
*E. quadrilineata* FOEVPRB58	S3*	09.823
*A. latus* FOEVPRB4	S4*	09.688
*A. latus* FOEVPRB11	S5*	09.785

*Represents the S1–S5 peaks of Aspergillus STC at their retention times (Figures 11–15).

## Discussion

The culinary preparations in India place a significant emphasis on the use of various spices (Siruguri and Bhat, 2015). Ever since the beginning of time, people have been seasoning their food with various spices. It is well known that *F. vulgare* possesses pharmacological activities such as antibacterial, anti-inflammatory, apoptotic, and anticancer activities, among others. Despite these diverse roles, fennel is susceptible to mycological degradation, continually being colonized by mycoflora like *Aspergilli*, *Curvularia*, and *Fusaria* (Mahata et al., 2022) during pre-harvest procedures, post-harvest losses in the field and storage. The occurrence of *Aspergillus* species on *F. vulgare* is the first sign of possible mycotoxin exposure. Specifically, in India, fennel seeds are a widespread spice used by the populace, which raises the risk of mycotoxin consumption and adds to health hazards and complications. The study is the first of its kind to detect the toxigenic gene clusters using DNA-based assays in addition to STC detection and purification. A total of 14 different types of *Aspergilli* have been isolated and reported from *F. vulgare* (Mahata et al., 2022), and yet, another study reported mycotoxigenic *Aspergilli* produced at varying humidities and storage periods (Amadi and Adeniyi, 2009) to produce AFs, ochratoxins, and fumonisins. This seems to be the issue with the toxigenic potential of these species and environmental exposures that cause toxicity.

Mycotoxin contamination of seed crops caused by *Aspergillus* spp. has been a challenge in all types of agricultural commodities for a long time. Generally speaking, most spices appear to be susceptible to fungal infection and consequently mycotoxin contamination. Aflatoxin and STC contamination of grain crops is a matter of concern as these mycotoxins are carcinogenic in nature (Anonymous, 2013) and have been detected in a variety of agricultural products (Kang et al., 2022). Section *Nidulantes* encompasses numerous species like *A. nidulans*, *A. quadrilineatus*, *A. latus*, and *A. rugulosus* that are routinely encountered in the ecosystem, primarily inhabiting the soil or decomposing plant debris (Domsch et al., 2007). Closely related species of *A. nidulans*, *A. versicolor*, and certain *A. sydowii* isolates have often been isolated from stored grains, nuts, seeds, dried medicinal herbs, and other foods and feeds (Pitt and Hocking, 2009; Hong et al., 2015). In addition, homothallic species of *A. nidulans*, *A. quadrilineatus*, *A. spinulosporus* (*Emericella echinulata*), and *A. rugulosus* have been mostly implicated in invasive infections (Yu et al., 2013; Uhrin et al., 2015). In individuals with chronic granulomatous illness, *A. nidulans* is more prevalent and virulent than in other immunocompromised patients (Segal, 2009). *A. nidulans* and *A. quadrilineatus* have also been linked to sinusitis, endophthalmitis, and onychomycosis (Hubka et al., 2012; Sharma et al., 2015). Cases of aspergillosis have been reported in dogs and horses (Seyedmousavi et al., 2015), but these diseases are rather rare in animals.

Ascomycete genomes encode an average of 40 biosynthetic gene clusters that are essential for secondary metabolites (SM) biosynthesis (Pusztahelyi et al., 2015). Genomic investigations of *Aspergilli* indicate that each species contains about 39 and 80 biosynthesis gene clusters (Inglis et al., 2013). The gene cluster required for STC biosynthesis in *A. nidulans* (Brown et al., 1996) has been identified and is known to share similarities with those of *Podospora anserina* (Slot and Rokas, 2011).

The generation of natural products as a result of the secondary metabolism of microbes and plants is affected by a variety of environmental factors, namely, temperature, humidity, light, pH, and the source of nutrients, among other parameters. Consequently, this has been described as temperature and nitrogen supply influence AF production in *A. parasiticus* and STC synthesis in *A. nidulans*, but in distinct ways (Feng and Leonard, 1998).

The fungal isolates used in our experiment were chosen due to the fact that there is a serious cause for concern regarding their toxigenicity and STC production. STC-producing *Aspergilli*, namely *A. latus* (02 isolates), *E. quadrilineata* (02 isolates), and *A. nidulans* (01 isolate) were isolated from the *F. vulgare*. Identification of the various species of *Aspergillus* is an arduous task and has become even more challenging as a result of the close species association. Data from the selective media used for *Aspergillus* species cultivation, mycotoxin processing, and various molecular tools are brought together here for our better understanding. In our analysis, *Aspergilli* characterization was derived from a combination of detailed morphology (Figures 1–3) and molecular techniques (Table 1) that is composed of two DNA barcode genes, ITS and β-tubulin.

Fungi from the *Nidulantes* section are commonly present in foods and feeds destined for human and animal consumption. Though some isolates produce STC, not all isolates are producers, and this has motivated researchers to use screening methods to identify fungal isolates that can produce mycotoxins. In these processes, culture conditions with additives are used to enhance the growth and detect fluorescence that is potential STC producers. Once fluorescence was detected under UV light on the fourth day of incubation, it seemed as a preliminary determination of the toxigenic potential and unmistakably decisive for every sterigmatocystin isolate tested. Further, the addition of 2% β-cyclodextrin to the basal medium improved the efficiency of fluorescence and STC detection (Figure 9) on the fungal culture plates. Also, it has been established through well-known studies that genes involved in secondary metabolite production tend to cluster in fungal genomes (Keller and Hohn, 1997). Formerly, methods for determining the extent of actively transcribed loci included northern blotting or reverse transcription-polymerase chain reaction (RT-PCR) and evaluating the expression in secondary metabolite-inducing vs. non-inducing settings. This strategy is viable for individual clusters, as demonstrated for trichothecene toxins (Kimura et al., 2003; Brown et al., 2004), lolitrem (Young et al., 2006), fumonisins (Proctor et al., 2003), gliotoxin (Gardiner and Howlett, 2005), sirodesmin (Gardiner et al., 2004), and others.

The AF and STC biosynthesis pathways are primarily regulated by the *aflR* gene (regulatory gene for aflatoxin biosynthesis) in *A. flavus*, *A. parasiticus*, and *A. nidulans* (Yu et al., 1996b; Yu and Keller, 2005). The *A. nidulans aflR* gene cluster is comprised of approximately 24 additional genes that are directly or indirectly involved in the production of the AF-related mycotoxin STC. It has been revealed that the protein known as *A. nidulans* AflR (AnAflR) interacts with the promoter regions of a number of AF and STC cluster genes (*stc* genes) (Fernandes et al., 1998). Moreover, it was demonstrated that the *laeA* gene, which is responsible for the loss of *aflR* expression, is implicated in the universal regulation of AF, STC, penicillin, and gliotoxin in numerous fungi.

A conserved filamentous fungus protein known as laeA (loss of *aflR* expression A) is also known as a conserved virulence factor in every pathogenic fungus that has been explored to this date (Wiemann et al., 2010; López-Berges et al., 2013). LaeA is a transcription factor that regulates the formation of gliotoxin, fumagillin, fumigatin, and helvolic acid metabolites in *A. fumigatus* (Keller et al., 2006). In *A. nidulans*, protein laeA, a potential methyltransferase, is a master regulator of secondary metabolism, which is essential for the expression of *aflR* and other secondary metabolite biosynthesis genes undergo chromatin remodeling (Bok et al., 2006; Patananan et al., 2013). LaeA expression is negatively controlled in a unique feedback loop by aflR, a STC transcription factor, as well as by two signaling components, protein kinase A and RasA (Bok et al., 2005; Sugui et al., 2007). They established that when *laeA* is deficient, secondary metabolism and pathogenicity are impaired. LaeA isolates contain hyphal pigments and play a minor role in spore production, indicating that its primary function is to regulate metabolic gene clusters (Bok and Keller, 2004). Additionally, laeA is necessary for the production of light-dependent conidia, which requires the veA protein to be in its native state (Sarikaya Bayram et al., 2010).

An important component of the circuit is pacC (pH-responsive transcription factor) (Tilburn et al., 1995), a zinc finger protein that activates alkaline-expressed genes while inhibiting the expression of acid-expressed genes. An alkaline-expressed gene in *A. nidulans*, isopenicillin N synthase (ipnA), has been used to study the binding of pacC to the promoter. In *A. nidulans*, a transcription factor called pacC modulates the expression of acidic and alkaline-structural genes based on the pH of the extracellular medium (Caddick et al., 1986; Tilburn et al., 1995). Sequential proteolysis of pacC forms the active form of the protein, PacC27, at an alkaline pH, which positively influences the activation of alkaline genes and negatively affects the regulation of acidic genes (Díez et al., 2002; Arst and Peñalva, 2003). Proteolysis of pacC during constitutive processing increases pathogenicity and promotes invasive development (Bignell et al., 2005). It demonstrates that both the pacC process and Pal-mediated pH signaling are essential for the promotion of pathological processes and that constitutive *pacC* activation promotes virulence (Bignell et al., 2005). The pathogenicity of *A. nidulans* is dependent on pH signaling and the pH-responsive transcription factor pacC (Bignell et al., 2005).

The *fluG* (fluffy) gene encodes a cytoplasmically localized protein that is maintained at relatively constant levels throughout the entire life cycle of *A. nidulans* (Lee and Adams, 1994), synthesizes an extracellular factor, and drives both development-specific processes and the stimulation of flbA (fluffy with low brlA expression), which then inhibits fadA (fluffy-autolytic dominant) signaling (Yu et al., 1996b). Researchers looking into the early events that lead to conidiation activation and STC synthesis in *A. nidulans* discovered six genes, *fluG* (Adams et al., 1992; Seo et al., 2006), and *flbA–flbE*, that are necessary for normal *brlA* activation (Wieser et al., 1994). Each one of these genes can be mutated to produce undifferentiated, fluffy colonies (Wieser et al., 1994). *A. nidulans fluG* mutants are incapable of producing the secondary metabolite STC (Hicks et al., 1997). The carbon and nitrogen sources, as well as the pH of the medium, are among the most critical physiological factors of AF/STC production in *Aspergilli.* In *A. parasiticus*, simple carbohydrates such as glucose and sucrose promote AF production, but in *A. nidulans*, STC is produced (Payne and Brown, 1998). Additionally, while ammonium promotes the biosynthesis of AF in *A. parasiticus*, it inhibits the formation of STC in *A. nidulans* (Guzman-de-Peña et al., 1998).

FlbA and FadA were the first RGS-Gα (regulator of G protein signaling-G protein α subunits) pair found in a filamentous fungus. Together, they are essential for the upstream control of hyphal growth, maturation, and secondary metabolite biosynthesis (Yu et al., 1996a; Yu and Keller, 2005). When *flbA* function is impaired, a fluffy-autolytic phenotype similar to that generated by FadA^*d*+^ (fluffy-autolytic phenotype) mutant alleles is produced (Yu et al., 1996a). A first *A. nidulans* regulator of G protein signaling (RGS) protein, *flbA*, was identified by the analysis of a fluffy-autolytic mutant. FlbA and FadA are necessary for the expression of *aflR*, which encodes a transcription factor specific to the STC biosynthetic pathways. Conidiation is genetically connected to STC biosynthesis in *A. nidulans* (Hicks et al., 1997). When the *flbA* gene is deleted, the *aflR* and *brlA* genes, as well as the consequent STC synthesis and conidiation, are all negatively regulated (Hicks et al., 1997).

Two fatty acid synthases (FAS) enzymes and polyketide synthase (pksA) combine to make the polyketide from a hexanoyl beginning unit (NR-PKS, PksA). It was discovered that fatty acid and polyketide synthases are responsible for controlling the early steps of AF production (Watanabe et al., 1996; Watanabe and Townsend, 2002). In *A. nidulans*, STC production was also found to be dependent on FASs, which were named *stcJ* and *stcK* (Brown et al., 1996). Gene disruption experiments on *A. parasiticus* revealed that AF synthesis requires the *pksA* genome (Chang et al., 1995).

Mutagenesis screening for novel VeA-dependent STC regulators in *A. nidulans* led to the discovery of the nucleus-based *mtfA* (master transcription factor A) transcription factor. A recent transcriptome investigation of *mtfA* in *A. nidulans* and *A. fumigatus* revealed its role in controlling the expression of hundreds of genes, including secondary metabolite gene clusters. In *A. nidulans*, *mtfA* regulates the production of STC, penicillin, and terriquinone A (Ramamoorthy et al., 2013), and other gliotoxins in *A. fumigatus.* This regulatory section includes clusters essential for mycotoxin production (Lind et al., 2015), some of which are known virulence factors (Coméra et al., 2007; Kwon-Chung and Sugui, 2009). MtfA exerts its effect on STC synthesis by modulating the expression of the STC gene cluster activator *aflR*. Along with affecting secondary metabolism, *mtfA* also has an impact on the development of *A. nidulans* both asexually and sexually. Additionally, *mtfA* is needed to maintain the proper development of sclerotia during conidiation.

Seven STC-linked genes were detected in the isolates tested (Figures 4–8) in this investigation, indicating that these isolates had positive amplification patterns for the STC gene cluster (Table 4). With a flow rate of 1 mL min^–1^ during HPLC analysis, the STC retention times (t_*R*_) for the *A. nidulans* FOEVPRB45, *E. quadrilineata* FOEVPRB49, *E. quadrilineata* FOEVPRB58, *A. latus* FOEVPRB4, and *A*. *latus* FOEVPRB11 isolates were 10.120 (Figure 11), 9.982 (Figure 12), 9.823 (Figure 13), 9.688 (Figure 14), and 9.785 (Figure 15) min. Quantification was accomplished by analyzing the peak areas of the STC and comparing them to those of the standard (Supplementary Figure 1); in this particular instance, STC employed for commercial purposes was purchased. The STC was detected and confirmed from five different isolates of *Aspergillus* using the HPLC-UV method. We were able to achieve separate STC peaks as shown in the chromatograms (Figures 11–15). Consequent to HPLC analysis, all five isolates tested positive and were evaluated for their potential ability to produce STC under optimal conditions of fungal growth. The HPLC-UV detection method was utilized in a prior experiment that was carried out by Marley et al. (2015) to look for STC contamination in a variety of foods and beverages, namely, cereals, seeds, livestock feed, dairy, and beer. This particular study reported HPLC chromatograms of STC, with the UV detector configured to 325 nm. STC was proven to have been detected in two samples of light and dark beer by the HPLC-UV analysis, and the presence of STC in the samples was validated by the HPLC chromatograms. This discovery was made in a study that was published in Veršilovskis et al. (2008). The level of STC found in beer was proportional to the amount found in barley. A first and effective HPLC-UV approach for determining STC production by *Aspergilli* is suitable for the efficient, specific, and routine analysis of STC production.

There are only a few accounts of STC occurring spontaneously, despite the fact that fungi capable of making STC are ubiquitous all over the world. In a focused study by Tabata (2011), STC was not detected in harvested grains or cheese but was found to be present in preserved products. STC has been detected in oregano, up to 28.0 μg/kg from Turkey, in thyme up to 14.0 μg/kg from Poland (Reinholds et al., 2017), and in paprika, 18.0 μg/kg from South Africa (Motloung et al., 2018). In another investigation, moderate quantities of STC in black pepper and chili were detected in Sri Lanka at 49.0 and 32 μg/kg, respectively (Jacxsens and De Meulenaer, 2016). The discovery of these potentially toxic variants may point to a possible presence of STC contamination of fennel grown in India. The data suggest an enormous danger of STC contamination in fennel, which can be worsened by unscientific methods, poor technologies applied by small farmers, and lack of temperature and humidity control during storage. Nonetheless, *Aspergillus* species from the *Nidulantes* Section are found in the Indian fennel analyzed in this study, highlighting the necessity to monitor or implement measures to limit the population’s STC intake. Accurate and timely identification of toxigenic fungi and their respective toxin may open doors for further research on STC contamination in fennel grown in India. The study could serve as a template for further research inquiries spanning different food groups. To this day, there has been no research conducted on the sterigmatocystin potential of *Aspergillus* produced from *F. vulgare*.

## Conclusion

In conclusion, the various outcomes of our study indicated that five *Aspergillus* isolates from the *Nidulantes* Section isolated from *F. vulgare* in India were STC producers. This demonstrated that fennel spice could harbor carcinogenic compounds, in particular STC. Therefore, the potential cumulative toxicities and synergistic action from these mycotoxins are major concerns in terms of risk assessment. This report gives a preliminary evaluation of the degree to which the spices may be contaminated with *Aspergilli*, as well as their vulnerability to fungal infection and STC production. In future, more research with larger, diverse samples of fennel, and other foodstuffs should be conducted to ascertain the contamination levels of STC and other possible mycotoxins present in spices, as well as their associated consumer concerns. After harvest, advancements in drying and storing procedures might restrict fungal contamination and mycotoxin exposure, but consumers’ safety also relies on suitable storage environments and quality standards at every stage of processing, packaging, or marketing. In order to limit the risk of disease in humans and animals in complex food chains, we emphasize the significance of reliable tests and strict government policies and regulations on permissible levels of mycotoxin, especially for spices.

## Data availability statement

The datasets presented in this study can be found in online repositories. The names of the repository/repositories and accession number(s) can be found below: https://www.ncbi.nlm.nih.gov/genbank/ (MN791101; MN791104; MN791105; MN791110; and MN791111).

## Author contributions

PM conceptualized, designed, performed, executed the research experiments and written the manuscript. RD conceptualized, guided the research experiments and written the manuscript. LG and PT helped in manuscript preparation and assisted in experimentation. All authors contributed to the article and approved the submitted version.
