# Supplementary material for: First report on the metabolic characterization of Sterigmatocystin production by select Aspergillus species from the Nidulantes section in Foeniculum vulgare
Source: Front Microbiol. 2022 Aug 26;13:958424. doi: 10.3389/fmicb.2022.958424 (PMC9459157; doi:10.3389/fmicb.2022.958424)
Supplement: Supplementary file 1 [file Image_1.pdf]

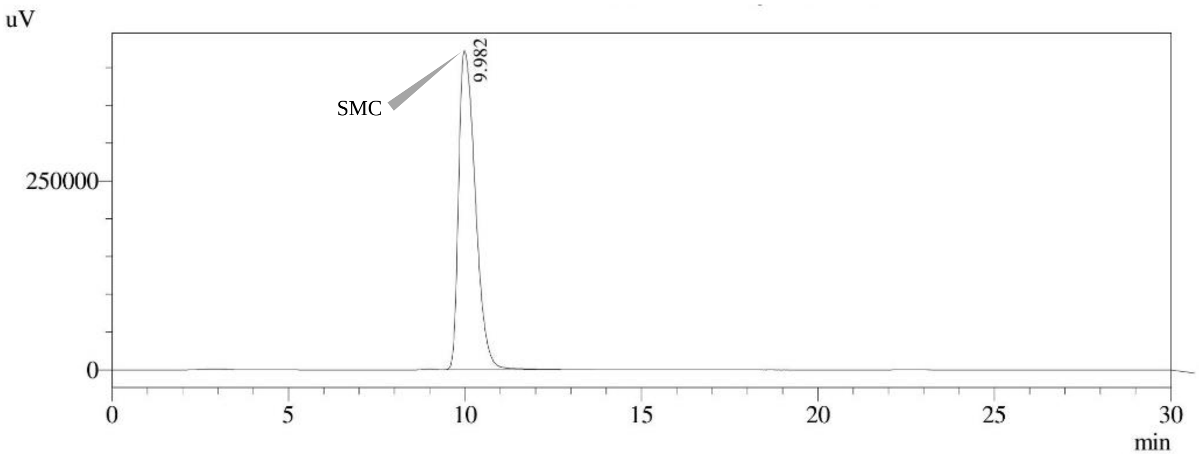

Supplementary Figure 1 The HPLC chromatogram of a sterigmatocystin standard UV peak at 325 nm and the retention time (t_R) of 9.982 min.
